# The association between social capital and quality of life among type 2 diabetes patients in Anhui province, China: a cross-sectional study

**DOI:** 10.1186/s12889-015-2138-y

**Published:** 2015-08-15

**Authors:** Fuyong Hu, Li Niu, Ren Chen, Ying Ma, Xia Qin, Zhi Hu

**Affiliations:** Hefei Second People’s Hospital, Hefei, China; School of Health Services Management, Anhui Medical University, No. 81, Meishan Road, Hefei, 230032 Anhui China; The First Affiliated Hospital of Anhui Medical University, Hefei, China

## Abstract

**Background:**

To investigate the association between social capital and quality of life among type 2 diabetes patients in Anhui province, China.

**Methods:**

In a cross-sectional study, 436 adults with type 2 diabetes were interviewed. The two domains of Quality of life, physical component summary (PCS) and mental component summary (MCS), were measured using the Short-Form Health Survey (SF-36). A modified instrument scale was used to measure cognitive and structural social capital. Multiple logistic regression models were used to assess the associations between social capital and quality of life, adjusting for social economic status and risk factors for health.

**Results:**

24.3 % of participants (106) were in poor PCS and 25.0 % (109) in poor MCS. The proportions of participants who had low cognitive and structural social capital were 47.0 % (205) and 64.4 % (281), respectively. Results of logistic regression models showed that cognitive social capital was positively associated with PCS (OR = 1.84; 95 % CI: 1.12, 3.02) and MCS (OR = 1.65; 95 % CI: 1.03, 2.66). However, the associations between structural social capital and PCS (OR = 0.80, 95 % CI: 0.48, 1.34) and MCS (OR = 0.62; 95 % CI: 0.38, 1.01) were not statistically significant.

**Conclusions:**

It is the first study in China to investigate associations between quality of life and social capital in type 2 diabetes. Findings document that cognitive social capital is associated with the quality of life of type 2 diabetes patients. Our study suggests that the social capital theory may provide a new approach to increase physical resources in diabetes prevention and control, especially in Low and Middle Income countries (LMICs).

**Electronic supplementary material:**

The online version of this article (doi:10.1186/s12889-015-2138-y) contains supplementary material, which is available to authorized users.

## Background

China in the recent years has the largest number of persons suffering from type 2 diabetes (diagnosed or undiagnosed) in the world. Type 2 diabetes patients are widely distributed in China, and the diabetes national prevalence is estimated at as of 9.32 % in 2014 [1]. Diabetes patients suffer from many complications (heart, brain, kidney, peripheral nerves, eye and foot injury induced by marcovascular and microvascular damage) that reduce the quality of their life [2, 3]. Given that diabetes is a chronic disease and cannot be cured, improvement or maintenance of adequate quality of life is one of the most important public health challenges to both developing and developed nations [4, 5]. Previous studies have documented that it’s multi-faced and not multi-faced factors influence quality of life, including the stage of the disease [6], life style [7], access to medical services [8], and social economic status [9]. However, few studies have investigated the determinants of quality of life among Chinese diabetes patients [10, 11]. Culture that has been considered an ecological-level factor influencing mental processes, human behaviors and health, may also influence quality of life [12]. Different from the western countries where individualist culture prevails, the Chinese culture is more collectivist. Individuals with collectivist cultures tend to seeking for social support and maintain social status in the social structure in which they live [13]. According to the Chinese culture, diabetes patients may buffer physical and mental suffering of diabetes by reconstructing their social networks and obtaining material and emotional social support from their social networks.

Social capital has been regarded as an important invisible health resource [14]. With growing recognition of the social determinants of health and numerous empirical studies, largely conducted in Western societies, social capital is an increasingly important construct in healthcare [15–17]. Social capital is defined as “features of social organization, such as trust, norms and networks, which can improve the efficacy of society by facilitating coordinated actions” [18]. Aiming at minimizing survival risk, social capital is the produce of human socialization, and the result of conscious or unconscious human investment strategy [19]. Social capital produces effects on human health through the following approaches, diffusion of knowledge about health promotion, maintenance of healthy behavior, access to healthcare services and amenities, acquirement of emotional or material support, and maintenance of mutual respect in social networks [15]. Holtgrave et al. examined the association between social capital (Putnam’s measure) and the prevalence of obesity and diabetes at the state level in USA, and concluded that greater levels of social capital might be protective against obesity and diabetes [20]. Years later, the protective effects of social capital on glucose control of diabetes have been found, suggests that social capital may improve symptom of diabetes [21, 22]. Social capital may be more important in the Chinese culture because the general nature of guanxi, which is similar to social capital, plays an important impact on social and economic relationships [23]. Chinese adult are more likely to establish relationship through the geographical and kinship relations to fulfill similar functions and collective benefits, especially old people [24]. However, no studies have been conducted in China to assess the association between social capital and quality of life among diabetes patients. The objectives of this study were to describe the relationship between social capital and the quality of life among type 2 diabetes patients in Anhui province, China. We hypothesized that social capital was positively associated with the quality of life in type 2 diabetes patients.

## Methods

### Ethics statement

Ethical approval for the study was obtained from the Biomedical Ethics Committee, Anhui Medical University.

### Study population and data collection

Based on the geographic distribution and economic level, we selected three cities in Anhui province: Fuyang-north, lower economic level; Hefei-central, high economic level; and Tongling-south, middle economic level [25]. We selected the respondents by simple random sampling (random number according to the healthcare record number) in each city. The total sample size was evaluated by the formula: *n* = Z^2^_α/2_(1 - P) / ε^2^*P* = 385, α = 0.05, ε = 0.10, Z_α/2_ = 1.96, *P* = 0.5. Inclusion criteria included individuals who were diagnosed with type 2 diabetes, aged 18 or older, living at home, and did not have cognitive impairment and disability. A convenience sample of 446 adults with type 2 diabetes (153 in Fuyang city, 154 in Hefei city, and 139 in Tongling city) was recruited for participation in this study.

Cross sectional surveys were conducted in these cities between August and October, 2014. All of the eligible respondents were identified from the chronic diseases database at the three local Centers for Disease Control and Prevention (CDC). With assistance from CDC staff, respondents were interviewed face-to-face by trained investigators from the Anhui Medical University after they expressed a verbal understanding of the purposes and procedures of the study and signed consent forms. The data collection took about 30 min. Each respondent received about a dollar’s (6 RMB) worth of gift as compensation for their time after the interview.

### Assessment of quality of life

The Short-Form Health Survey (SF-36) is one of the most widely used instruments for evaluating people’s quality of life [26]. The Chinese-translated version of the SF-36 questionnaire has previously been used and verified as a scale with high reliability and validity for the measurement of Quality of life among older Chinese adults with diabetes [27, 28]. In this study, Quality of life of respondents was evaluated using this Chinese version. SF-36 contains 8 dimensions, including physical function, role physical, bodily pain, general health, vitality, social functioning, role emotion, and mental health. These 8 dimensions can be simplified into physical component summary (PCS) and mental component summary (MCS), reflecting physical health and mental health respectively. PCS and MCS scores were assessed and calculated by T-score transformations [28]. After that, each respondent’s PCS and MCS scores were dichotomized by the cutoff point of the first quartile of PCS and MCS scores: scores lower than first quartile scores meant poor quality of life [29].

### Social capital measurement

Social capital has been measured via individual cognition and behavior in the health field. It can be divided into cognitive and structural social capital which may have different effects on health outcomes, even though the controversial about its measurement remains [30–32]. Structural social capital includes extent and intensity of associational links or activity, and cognitive social capital covers interpersonal trust, reciprocity, social support and cohesion [30]. This study adopts a perspective of cognition and structure on social capital. According to the World Bank’s Social Capital Assessment Tool and the related literature, six dimensions of social capital were considered in this study: social participation, social networks, social support, trust, reciprocity and cohesion [33, 34]. We selected some commonly used items corresponding to the six dimensions and adapted them to the Chinese context. The social capital questionnaire was reported in Additional file 1 and had been attached to the last.

First, six dimensions were summed by the scores of corresponding items. Second, cognitive social capital and structural social capital were measured by producing a component score of six dimensions using factor analysis. Finally, each respondent’s cognitive and structural social capital scores were dichotomized by the cutoff point of the mean component: high cognitive and structural social capital (component score ≥0), low cognitive and structural social capital (component score <0 [33]).

### Socio-economic status (SES) and risk factors

SES contained information of residency (rural vs. urban), gender, age, ethnicity, marital status, education level, main occupation and monthly income. Risk factors contained current smoking, participation in a moderate-intensity physical activity (150 min per week), two-week prevalence of any diseases, and comorbidity of other chronic diseases [10, 35, 36].

### Data analysis

Descriptive statistics was performed on the sample, and the results were expressed as mean ± standard deviation (SD) or percentage. Cronbach’s alpha values were calculated and used to evaluate the reliability of social capital scale in reliability analysis. Scores of social capital were calculated in factor analysis. Crude odds ratios (ORs) and 95 % confidence intervals (95 % CI) were calculated in order to analyze associations between Quality of life and social capital by binary logistic regression (model 1). The adjusted ORs measuring the association were estimated by controlling for SES variables (model 2) and both SES and risk factors (model 3). The statistical analysis was performed using the SPSS statistical package (Windows version 16.0, SPSS Inc., Chicago, Illinois), and *p* ≤ 0.05 was taken as the statistically significant level.

## Results

A total of 436 respondents completed the questionnaire, with a respondent rate of 97.8 %. The overall Cronbach’s alpha coefficient for social capital was 0.87, and for each dimension Cronbach’s alpha coefficient was 0.79 (social participation), 0.62 (social networks), 0.75 (social support), 0.91 (trust), 0.63 (reciprocity) and 0.87 (cohesion). The Cronbach’s alpha coefficient was 0.88 for cognitive social capital and 0.79 for structural social capital.

Table 1 present descriptive information about the study sample, the respondents had a mean age of 67.1 ± 10.2 years (range, 33–96 years), most of whom were female (67.7 %). The majority of respondents were Han ethnicity (98.9 %) and married (85.1 %). About two thirds (65.8 %) had low income (<1000 RBM/month). A large proportion of respondents (71.8 %) participated in the moderate-intensity physical activity per week, did not smoke (74.5 %), and were not ill over the past two weeks (78.2 %). A little more than half of respondents had other chronic diseases (53.7 %), including cardiovascular diseases, cancer, chronic respiratory diseases. The mean scores of PCS and MCS were 67.4 and 64.9, respectively. Based on the cutoff points for PCS (51.9) and MCS (67.4), 106 (24.3 %) participants were in poor PCS and 109 (25.0 %) in poor MCS. The proportions of respondents who had low cognitive and structural social capital were 47 % (205) and 64.4 % (281), respectively.

**Table 1:** Characteristics of the study respondents (*N* = 436)

Variables		No. or mean	Percentage or SD
Census register	Rural	224	51.4
Gender	Male	141	32.3
Age (year)		67.1	10.2
Ethnicity	Han	431	98.9
Marital status	Currently married	371	85.1
	Widowed/Single	65	14.9
Education level	Illiterate	188	43.1
	Primary	102	23.4
	Junior high	88	20.2
	Senior high	39	8.9
	College or above	19	4.4
Occupation	Farmer	221	50.7
	Employee	182	41.7
	Non-working	33	7.6
Monthly income	<1000	287	65.8
(RMB)	1000 ~ 2000	99	22.7
	≥2000	50	11.5
Current smoking	No	325	74.5
Moderate-intensity physical activity	No	123	28.2
Two-week prevalence	No	341	78.2
Other chronic diseases	No	202	46.3
PCS		67.4	21.0
MCS		64.9	15.3
Cognitive social capital	Low	205	47.0
Structural social capital	Low	281	64.4

The results of logistic regression are showed in Table 2. Cognitive social capital was significantly associated with PCS and MCS. In model 1, diabetes patients with higher cognitive social capital had higher odds of high PCS (OR = 2.15, 95 % CI: 1.38, 3.37) and MCS (OR = 1.97, 95 % CI: 1.27, 3.07). After adjusting for SES and risk factors, cognitive social capital was still significantly associated with PCS and MCS in model 2 and 3. The ORs for high structural social capital compared to high PCS and MCS level were not significant in all three models.

**Table 2:** Cognitive and structural social capital linked with PCS and MCS (*N* = 436)

		Model 1 OR (95 % CI)	Model 2 OR (95 % CI)	Model 3 OR (95 % CI)
PCS				
Cognitive social capital	Low	1.00	1.00	1.00
	High	2.15 (1.38–3.37)***	1.93 (1.21–3.09)**	1.84 (1.12–3.02)*
Structural social capital	Low	1.00	1.00	1.00
	High	1.04 (0.66–1.64)	0.87 (0.54–1.42)	0.80 (0.48–1.34)
MCS				
Cognitive social capital	Low	1.00	1.00	1.00
	High	1.97 (1.27–3.07)**	1.70 (1.07–2.69)*	1.65 (1.03–2.66)*
Structural social capital	Low	1.00	1.00	1.00
	High	0.84 (0.54–1.32)	0.92 (0.50–1.67)	0.62 (0.38–1.01)

Model 1: Unadjusted. Model 2: Adjusted for SES. Model 3: Adjusted for SES and risk factors

*: p <0.05, **: p <0.01, ***: p <0.001

## Discussion

This study was to investigate the association between social capital and quality of life among type 2 diabetes patients in Anhui province, China. Findings of this study document that the levels of both quality of life and social capital were relatively low among diabetes patients, but strong positive associations between quality of life and social capital. Given that diabetes patients suffer long-term physical and emotional complications of type 2 diabetes, our results may provide important information regarding improvement of quality of life via the enhancement of social capital. To our knowledge, it is the first study in China to investigate such associations in type 2 diabetes patients.

Influenced by the social environment, awareness and treatment among Chinese diabetes patients is less than the western countries [37]. There are deficiencies in treatment and management of Chinese type 2 diabetes patients, including low ratio of community health service staff to patients, insufficient service and public health funding, and limited access to medical services [38]. These deficiencies may reduce quality of life among Chinese type 2 diabetes patients, and lead to a higher rate of mortality [1].

We selected items for six dimensions of social capital according to the related literature. Scores of social participation were on the low side, indicating that behavior of social participation may be certain differences between mainland China and western countries. Formal organizations (such as sports association and religious association) defined in western countries are rare in China. Culture of association is not popular in mainland China, especially those non-government organizations. We considered that individual level social capital is only accumulated by individual’s social networks (guanxi) and interpersonal norms in the majority of Chinese residents.

Previous studies showed that social capital was positively associated with quality of life in the elderly [39–41], adults [42], long-term social assistance [43], patients with fibromyalgia [44], multiple sclerosis patients [45], women [46, 47], and AIDS patients [33]. In this cross-sectional study, we found some consistent evidence to support the hypothesis that higher cognitive social capital was associated with higher PCS and MCS, the two domains of quality of life, after adjustment for SES and risk factors. Cognitive social capital indicates ability that individual can use to acquire social resources from family, community, medical services, and society. Patients with high cognitive social capital may actively seek for information, material, and emotional support networks, comply with social norms and peer control, trust and work closely with others in their daily activities, all of which could lead to receive adequate medical services and psychological support to buffer sufferings caused by diabetes [13].

The crude analysis indicated the association between quality of life and structural social capital not significant. In this study, structural social capital was mainly composed of social participation. According to our study, the majority of respondents were rarely participating in formal organizations, such as politic parties, sports associations, religious and professional originations. The low level of participation in such organized activities may lead to low or non-association between this type of social capital and quality of life.

### Limitation

This study was subject to several limitations. First, the study population in this survey was a convenient sample, with low representativeness that may deviate from overall Chinese population. Second, because of the nature of the cross-sectional study, the relationship is just a pure association, and need more information to support the possible causal relationship. Third, because social capital used in this study was measured at the individual level, the impact of ecological level and the entire social capital or six different dimensions on Quality of life were not considered. Finally, other risk factors such as blood sugar control and diet habit were not included in the study, which may undermine the main findings.

## Conclusions

This study suggests that cognitive social capital may have an important protective role in improving the Quality of life of type 2 diabetes patients in Anhui province, China. This initial finding suggests that the social capital theory may provide a new idea to solve the shortage problem of physical resources in diabetes prevention and control, especially in Low and Middle Income countries (LMICs).

## Additional file

Additional file 1:
**Social Capital Assessment Tool.** (DOC 30 kb)

## Abbreviations

PCS: Physical component summary
MCS: Mental component summary
SF-36: The Short-Form Health Survey
CDC: Centers for Disease Control and Prevention
SES: Socio-economic status
SD: Standard deviation
ORs: Odds ratios
CI: Confidence intervals
SPSS: Statistical package for the social sciences
LMICs: Low and Middle Income countries

## References

[1] International Diabetes Federation, Diabetes Atlas. (2014). [http://www.idf.org/sites/default/files/Atlas-poster-2014_EN.pdf]. Accessed June, 28, 2015.

[2] Chin YR, Lee IS, Lee HY (2014). Effects of hypertension, diabetes, and/or cardiovascular disease on health-related quality of life in elderly Korean individuals: a population-based cross-sectional survey. Asian Nurs Res.

[3] Landman GW, van Hateren KJ, Kleefstra N, Groenier KH, Gans RO, Bilo HJ (2010). Health-related quality of life and mortality in a general and elderly population of patients with type 2 diabetes (ZODIAC-18). Diabetes Care.

[4] Chen L, Magliano DJ, Zimmet PZ (2012). The worldwide epidemiology of type 2 diabetes mellitus--present and future perspectives. Nat Rev Endocrinol.

[5] Danaei G, Finucane MM, Lu Y, Singh GM, Cowan MJ, Paciorek CJ (2011). National, regional, and global trends in fasting plasma glucose and diabetes prevalence since 1980: systematic analysis of health examination surveys and epidemiological studies with 370 country-years and 2.7 million participants. Lancet.

[6] Vallis M, Ruggiero L, Greene G, Jones H, Zinman B, Rossi S (2003). Stages of change for healthy eating in diabetes: relation to demographic, eating-related, health care utilization, and psychosocial factors. Diabetes Care.

[7] Brown DW, Balluz LS, Giles WH, Beckles GL, Moriarty DG, Ford ES (2004). Diabetes mellitus and health-related quality of life among older adults. Findings from the behavioral risk factor surveillance system (BRFSS). Diabetes Res Clin Pract.

[8] Bajaj S, Jawad F, Islam N, Mahtab H, Bhattarai J, Shrestha D (2013). South Asian women with diabetes: psychosocial challenges and management: consensus statement. Indian J Endocrinol Metab.

[9] Lounsbury DW, Hirsch GB, Vega C, Schwartz CE (2014). Understanding social forces involved in diabetes outcomes: a systems science approach to quality-of-life research. Qual Life Res.

[10] Cong JY, Zhao Y, Xu QY, Zhong CD, Xing QL (2012). Health-related quality of life among Tianjin Chinese patients with type 2 diabetes: a cross-sectional survey. Nurs Health Sci.

[11] Pan C, Yang W, Jia W, Weng J, Liu G, Luo B (2012). Psychological status of Chinese patients with type 2 diabetes: data review of diabcare-China studies. Diabet Med.

[12] Matsumoto D, Kouznetsova N, Ray R, Ratzlaff C, Biehl MRJ (1999). Psychological culture, physical health, and subjective well-being. J Gend Cult Health.

[13] Kleinman A, Wang WZ, Li SC, Cheng XM, Dai XY, Li KT (1995). The social course of epilepsy: chronic illness as social experience in interior China. Soc Sci Med.

[14] Hsieh CH (2008). A concept analysis of social capital within a health context. Nurs Forum.

[15] Murayama H, Fujiwara Y, Kawachi I (2012). Social capital and health: a review of prospective multilevel studies. J Epidemiol.

[16] Story WT (2013). Social capital and health in the least developed countries: a critical review of the literature and implications for a future research agenda. Glob Public Health.

[17] Fujisawa Y, Hamano T, Takegawa S (2009). Social capital and perceived health in Japan: an ecological and multilevel analysis. Soc Sci Med.

[18] Nyqvist F, Forsman AK, Giuntoli G, Cattan M (2013). Social capital as a resource for mental well-being in older people: A systematic review. Aging Ment Health.

[19] Nettle D, Pepper GV, Jobling R, Schroeder KB (2014). Being there: a brief visit to a neighbourhood induces the social attitudes of that neighbourhood. PeerJ.

[20] Holtgrave DR, Crosby R (2006). Is social capital a protective factor against obesity and diabetes? Findings from an exploratory study. Ann Epidemiol.

[21] Long JA, Field S, Armstrong K, Chang VW, Metlay JP (2010). Social capital and glucose control. J Community Health.

[22] Farajzadegan Z, Jafari N, Nazer S, Keyvanara M, Zamani A (2013). Social capital - a neglected issue in diabetes control: a cross-sectional survey in Iran. Health Soc Care Community.

[23] Qi X (2013). Guanxi, social capital theory and beyond: toward a globalized social science. Br J Sociol.

[24] Gustafson K, Baofeng H (2014). Elderly care and the one-child policy: concerns, expectations and preparations for elderly life in a rural Chinese township. J Cross Cult Gerontol.

[25] Statistics Bureau of Anhui province. [http://www.ahtjj.gov.cn/tjj/web/tjnj_view.jsp#]. Accessed June, 28, 2015.

[26] Yang Z, Li W, Tu X, Tang W, Messing S, Duan L (2012). Validation and psychometric properties of Chinese version of SF-36 in patients with hypertension, coronary heart diseases, chronic gastritis and peptic ulcer. Int J Clin Pract.

[27] Lam CL, Tse EY, Gandek B, Fong DY (2005). The SF-36 summary scales were valid, reliable, and equivalent in a Chinese population. J Clin Epidemiol.

[28] Hu J, Gruber KJ, Hsueh KH (2010). Psychometric properties of the Chinese version of the SF-36 in older adults with diabetes in Beijing, China. Diabetes Res Clin Pract.

[29] Khedmat H, Karami GR, Pourfarziani V, Assari S, Rezailashkajani M, Naghizadeh MM (2007). A logistic regression model for predicting health-related quality of life in kidney transplant recipients. Transplant Proc.

[30] Harpham T, Grant E, Thomas E (2002). Measuring social capital within health surveys: key issues. Health Policy Plan.

[31] Lochner K, Kawachi I, Kennedy BP (1999). Social capital: a guide to its measurement. Health Place.

[32] Bertotti M, Watts P, Netuveli G, Yu G, Schmidt E, Tobi P (2013). Types of social capital and mental disorder in deprived urban areas: a multilevel study of 40 disadvantaged London neighbourhoods. PLoS One.

[33] Ma Y, Qin X, Chen R, Li N, Chen R, Hu Z (2012). Impact of individual-level social capital on quality of life among AIDS patients in China. PLoS One.

[34] Veenstra G, Luginaah I, Wakefield S, Birch S, Eyles J, Elliott S (2005). Who you know, where you live: social capital, neighbourhood and health. Soc Sci Med.

[35] Venkataraman K, Wee HL, Leow MK, Tai ES, Lee J, Lim SC (2013). Associations between complications and health-related quality of life in individuals with diabetes. Clin Endocrinol (Oxf).

[36] Elsawy B, Higgins KE (2010). Physical activity guidelines for older adults. Am Fam Physician.

[37] Steinman RA, Birshtein BK (2007). Treatment and awareness of type 2 diabetes in Beijing, China, compared to New York. Diabetes Educ.

[38] Xin C, Ge X, Yang X, Lin M, Jiang C, Xia Z (2014). The impact of pharmaceutical care on improving outcomes in patients with type 2 diabetes mellitus from China: a pre- and postintervention study. Int J Clin Pharm.

[39] Nilsson J, Rana AK, Kabir ZN (2006). Social capital and quality of life in old age: results from a cross-sectional study in rural Bangladesh. J Aging Health.

[40] Deshmukh PR, Dongre AR, Rajendran K, Kumar S (2015). Role of social, cultural and economic capitals in perceived quality of life among old age people in kerala, India. Indian J Palliat Care.

[41] Lucumi DI, Gomez LF, Brownson RC, Parra DC (2015). Social capital, socioeconomic status, and health-related quality of life among older adults in bogota (Colombia). J Aging Health.

[42] Kim D, Kawachi I (2007). U.S. state-level social capital and health-related quality of life: multilevel evidence of main, mediating, and modifying effects. Ann Epidemiol.

[43] Wahl A, Bergland A, Loyland B (2010). Is social capital associated with coping, self-esteem, health and quality of life in long-term social assistance recipients?. Scand J Caring Sci.

[44] Boehm A, Eisenberg E, Lampel S (2011). The contribution of social capital and coping strategies to functioning and quality of life of patients with fibromyalgia. Clin J Pain.

[45] Rimaz S, Mohammad K, Dastoorpoor M, Jamshidi E, Majdzadeh R (2014). Investigation of relationship between social capital and quality of life in multiple sclerosis patients. Glob J Health Sci.

[46] Salehi A, Harris N, Coyne E, Sebar B (2014). Trust and quality of life: a cross-sectional study of young women. Int J Soc Psychiatry.

[47] Sone K, Nakao M, Lamaningao P, Sugiura Y, Yamamotol H, Yamaoka K (2014). Relationship between active information exchange and the quality of life (qol) of women living in Lao People’s Democratic Republic. Southeast Asian J Trop Med Public Health.

