# Supplementary material for: The association between social capital and quality of life among type 2 diabetes patients in Anhui province, China: a cross-sectional study
Source: BMC Public Health. 2015 Aug 15;15:786. doi: 10.1186/s12889-015-2138-y (PMC4542125; doi:10.1186/s12889-015-2138-y)
Supplement: Additional file 1: — Social Capital Assessment Tool. (DOC 30 kb) [file 12889_2015_2138_MOESM1_ESM.doc]

Additional file

Social participation (Examples), not participated in = 0, average member = 1, active member = 2, leadership = 3.

1.1 Political party (the Communist Party)

1.2 Sports association (basketball or Ping-Pong associations)

1.3 Professional association (technology, art or cultivation associations)

1.4 Religious association (Church, Buddhist or Taoism associations)

1.5 Volunteer organization

1.6 Hobby association (dancing, singing or playing cards associations)

1.7 Colleague gathering

1.8 Relatives gathering

1.9 Townsmen association

1.10 Alumni association

1.11 Other social organizations

A 5-point likert scale was used to measure (Strongly disagree = 1 disagree = 2, not sure = 3, agree = 4, strongly agree =5).

Social networks

2.1 Do you often contact with relatives or family?

2.2 Do you often contact with friends?

2.3 Do you know the surrounding neighbors?

2.4 Do you often visit neighbors?

Social support

3.1 Does anyone supply instrumental support when you get trouble?

3.2 Does anyone supply economical support when you get trouble?

3.3 Do any organizations supply instrumental support when you get trouble?

3.4 Do any organizations supply economical support when you get trouble?

Trust

4.1 Do you believe that most people can be trusted?

4.2 Do you believe that the majority of relatives or family can be trusted?

4.3 Do you believe that the majority of friends can be trusted?

4.4 Do you believe that the majority of neighbors can be trusted?

4.5 Do you believe that the majority of local hospital staff can be trusted?

4.6 Do you believe that the majority of community physicians can be trusted?

4.7 Do you believe that the majority of community or village resident committee staff can be trusted?

Reciprocity

5.1 Would you like to provide support for relatives or family who need help?

5.2 Would you like to provide support for neighbors who need help?

5.3 Would you like to provide support for friends who need help?

5.4 Would you like to provide support for strangers who need help?

5.5 Do you think most people would try to take advantage of you if they got a chance? (Score reverse transformation)

Cohesion

6.1 Do you believe that most people get along well in your community?

6.2 Do you care about your community?

6.3 Do neighbors care about your community?

6.4 If you had to move away, do you reluctant to move?
